# Chemistry must respond to the crisis of transgression of planetary boundaries

**DOI:** 10.1039/d2sc03603g

**Published:** 2022-09-13

**Authors:** Stephen A. Matlin, Sarah E. Cornell, Alain Krief, Henning Hopf, Goverdhan Mehta

**Affiliations:** Institute of Global Health Innovation, Imperial College London London SW7 2AZ UK s.matlin@imperial.ac.uk; International Organization for Chemical Sciences in Development 61 rue de Bruxelles B-5000 Namur Belgium; Stockholm Resilience Centre, Faculty of Science, Stockholm University Stockholm Sweden; Chemistry Department, Namur University B-5000 Namur Belgium; Institute of Organic Chemistry, Technische Universität Braunschweig Braunschweig D-38106 Germany; School of Chemistry, University of Hyderabad Hyderabad 500046 India

## Abstract

Recent assessments alarmingly indicate that many of the world's leading chemicals are transgressing one or more of the nine planetary boundaries, which define safe operating spaces within which humanity can continue to develop and thrive for generations to come. The unfolding crisis cannot be ignored and there is a once-in-a-century opportunity for chemistry – the science of transformation of matter – to make a critical difference to the future of people and planet. How can chemists contribute to meeting these challenges and restore stability and strengthen resilience to the planetary system that humanity needs for its survival? To respond to the wake-up call, three crucial steps are outlined: (1) urgently working to understand the nature of the looming threats, from a chemistry perspective; (2) harnessing the ingenuity and innovation that are central to the practice of chemistry to develop sustainable solutions; and (3) transforming chemistry itself, in education, research and industry, to re-position it as ‘chemistry for sustainability’ and lead the stewardship of the world's chemical resources. This will require conservation of material stocks in forms that remain available for use, through attention to circularity, as well as strengthening engagement in systems-based approaches to designing chemistry research and processes informed by convergent working with many other disciplines.

## Abstract 2

Chemistry, the science of transformation of matter, is fundamental to achieving sustainability through ensuring biogeochemical flows do not transgress planetary boundaries. This requires urgent changes in chemistry education, research and industry.

## Graphical abstract

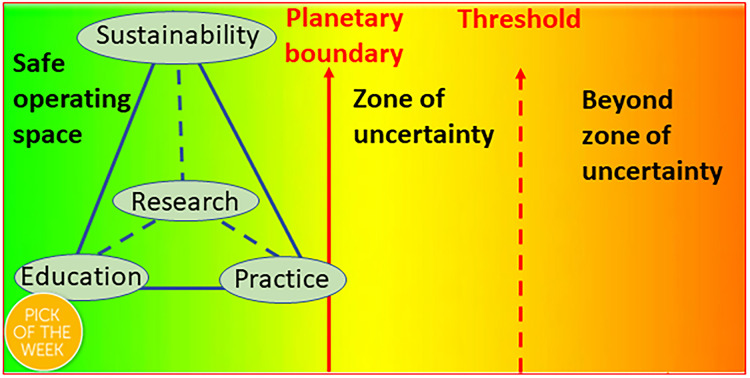

## Introduction

Chemistry's extraordinary success as the science of transformation of matter, bringing huge benefits to humanity, has been central to the expanding quantity and diversity of artefacts generated by human beings. The production of anthropogenic mass (global total dry mass of material contained in inanimate solid objects made by humanity) doubled every 20 years since 1900, in 2020 reaching 30Gt per year and totalling c. 1.1 trillion tonnes present on the planet.^1^ This accumulated amount equalled the dry weight of total biomass on Earth for the first time and, on current trends, total anthropogenic mass will be triple the dry weight of biomass by 2040. Concomitantly, the global chemical industry's production capacity (excluding pharmaceuticals) almost doubled between 2000 and 2017, from about 1.2 to 2.3 billion tonnes.^2^

However, there has been mounting environmental cost, including degradation of global habitat quality and loss of planetary ecosystem stability. Global atmospheric CO_2_ concentrations reached their highest historic level of 421.37 ppm in May 2022. They are likely to drive global warming since pre-industrial times above the 1.5 °C safe limit in the 2020s.^3^ At the same time, the majority of the 500 main chemicals on the market have been judged unsustainable in terms of their environmental impact.^4^

This is a wake-up call for all humanity to attend to the unfolding crisis. That it is not the first alarm heightens the urgency of now paying attention. Coining the term Anthropocene recognized that a transition was in rapid progress to a new period in which humanity has become the dominant force shaping the planet's environment.^5^ Harbingers of this transition^6^ were seen in writings on the vulnerability of ‘spaceship Earth’,^7^ the environment^8^ and the limits to growth.^9^ The 1987 World Commission on Environment and Development^10^ advanced the concept of sustainable development, a key step towards the UN Sustainable Development Goals (SDGs) agreed in 2015.^11^

As well as setting the SDGs, other global responses have included a range of efforts by the UN Environment Programme^12^ and Committee of the Parties (COP) negotiations of the UN Framework Convention on Climate Change.^13^ In all of these, roles for science and technology have been highlighted,^14^ the need for chemistry recognised and attention drawn to the mapping of SDGs directly into chemistry.^15^ Academia and industry have increasingly devoted attention to important aspects of the challenges,^16^ marked by developments including environmental chemistry since the 1950s and green chemistry which started to become prominent in the 1990s, as well as sustainable, one-world and circular chemistry. But much greater effort is now needed^17^ and chemistry needs to respond urgently and effectively across all branches of education, research and practice, with industry having centrally important roles.

How can chemists contribute to meeting these challenges and restore stability and resilience to the planetary system that humanity needs for its survival? To respond to the renewed and intensified ringing of alarms, and to develop the concept and apply the approach of chemistry for sustainability,^18^ three crucial steps are outlined in this article: (1) urgently working to understand the nature of the threats, from a chemistry perspective; (2) harnessing the ingenuity and innovation that are central to the practice of chemistry to develop sustainable solutions; and (3) transforming chemistry itself, in education, research and practice, to re-position it as a sustainability science and strengthen its capacities to work, in concert with other disciplines, on long-term approaches that will avoid unsustainable practices in the future.

## Understanding the problem

The planetary boundaries approach, developed in 2009–2015,^19^ identified nine aspects of anthropogenic threats to critical Earth systems, for each of which ‘safe operating spaces’ needed to be defined. For seven of these boundaries, control variables were proposed and threshold levels set for the safe operating limits. In many cases, the control variables chosen as indicators were chemical entities (Table 1), including the biogeochemical flows of carbon, nitrogen and phosphorus which are major contributors to currently accelerating planetary changes. The category of novel entities (including synthetic chemicals, microparticulate materials and other new types of engineered materials or organisms not previously known to the Earth system) is one of two planetary boundaries not yet officially quantified, owing to the difficulty of choosing control variables.

**Table 1:** Planetary boundaries and their chemical control variables at 2015

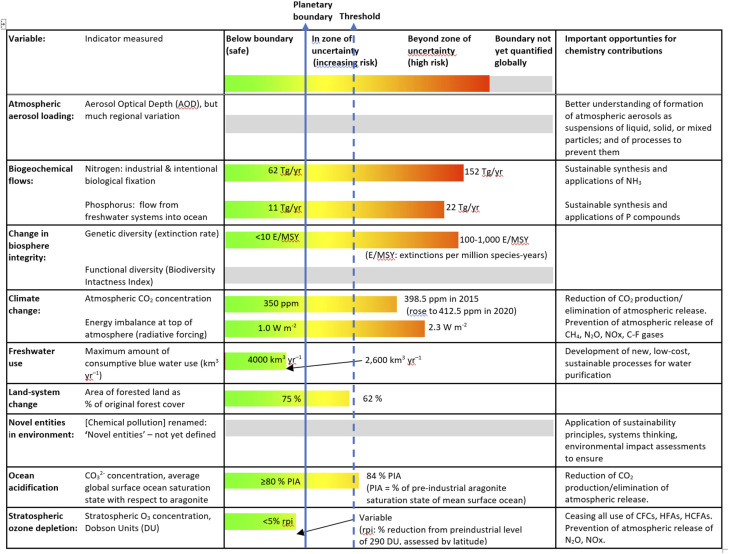

The handful of chemicals initially selected as signals of the planetary condition represent only the tip of a vast iceberg of materials that need to be considered. Around 200 million discrete chemical entities are known and the number grows at around 10 million per year. Many of these substances have only ever been prepared in sub-gram quantities, but over 350 000 chemicals have been registered for production and use, either as single entities or in a vast array of combination materials. Among ubiquitous devices, the smartphone typically contains at least 30 elements in an individual handset and different models of smartphones incorporate more than 70 of the periodic table's members, either in the elemental form or as compounds or mixtures.^20^ As well as the issue of depletion of useable elemental reserves, the impacts on the environment of these substances, when produced at large scale, remain largely unknown. The United Nations Environment Programme (UNEP) 2019 report, *Global Chemicals Outlook II*, showed that the commitment countries had made in 2002 at the World Summit on Sustainable Development, to minimize by 2020 the adverse effects of chemicals and pollutants passing into the environment, was not being achieved.^2^

Solutions to the challenges to sustainability presented by large-scale manufacturing are evolving – but this is still work in progress. The ‘throwaway’ society popularised in the 1950s–1960s was countered by growing concerns about over-consumption and environmental damage which led to a movement to ‘reduce, reuse and recycle’ (the ‘3Rs’). This alternative to the linear ‘take-make-dispose’ model has informed circular economy, waste reduction and sustainable consumption and production (‘living lightly’) policies in many countries.^21^

Two recent publications signal the urgency for chemistry to take action. One concerns the nature of the previously ill-defined planetary boundary related to ‘novel entities’. A measure of the production and release of plastics has been proposed as one possible parameter for this boundary, and the case presented that the safe operating space of the novel entities planetary boundary has already been transgressed.^22^ The other report^4^ has considered what planetary metrics are of relevance for the absolute environmental sustainability assessment of chemicals. Nearly 500 chemicals were assessed in relation to seven planetary boundaries and most were found to transgress at least one of the thresholds for their safe operating limits. The study has highlighted the need to incorporate absolute environmental sustainability criteria into environmental assessments for all chemical entities produced at large scale, to aid researchers and industries in the quest for sustainability.

## Chemistry for sustainable solutions

Sustainability science has developed a number of key insights relevant to all disciplines that seek to contribute to sustainable development. One principle is that sustainability is a property that emerges from the operation of an entire system and not simply attributable to isolated components. Thus, a product cannot be said to be ‘sustainable’ solely because it is derived from a renewable source, because its manufacturing process is based on green chemistry principles, or because it is recycled in some way after primary use, if it continues to be used in a manner or at a scale that transgresses planetary boundaries and damages Earth systems. It is now imperative that chemists developing new products or processes that will operate at manufacturing scale consider the potential environmental costs and impacts – not only for the core reactions or processes, but also for the entire chain of events from sourcing input materials and energy to the disposal of all by-products, wastes and the articles themselves at the end of the primary use. The consideration needs to include the entire gamut of short-to-long-term effects on all parts of the planetary environment, and to be concerned with the depletion of natural resources where these may become limiting – but even for the most abundant or renewable natural resources it needs to be concerned with how global scale-up and turnover of biogeochemical flows and the dispersal of materials across the planet may impact on these diverse aspects of the environment. While riding their hobby horses in pursuit of creative instinct or commercial interest, it is vital that chemistry practitioners carry out due diligence with regard to these considerations. All stages of supply, manufacturing, application and disposal need to be regarded using systems principles and tools. These are embedded in green, sustainable and circular chemistry, with quantifications derived from life cycle analysis, planetary boundaries and absolute environmental sustainability criteria. This totality of assessment provides the basis for overall judgement about sustainability as well as for the concept and approach encapsulated in ‘chemistry for sustainability’.^18^

### New reaction processes

Among the new and re-merging innovations currently receiving attention, those focusing on the sustainability of reaction processes, including the use of non-hazardous materials and conditions and applications of green solvents,^23^ will be of particular interest to synthetic chemists. The search for more environmentally benign processes for chemical reactions has branched into a number of directions in recent years. For the choice of reaction medium, at least three avenues have been under development. One is the identification of novel solvents, including ionic liquids, that offer economic and environmental advantages as well as chemical benefits.^24^ A second direction is the application of flow chemistry processes.^25^ The traditional approach to batch-wise chemical reactions in non-aqueous solvents presents many challenges in relation to the fate of solvents and the material and energy demands of synthesis, work-up and purification processes. Third, developments are advancing ‘solventless’ and ‘reagentless’ chemistry, including resurgent interest in mechanochemistry, microwave processes, sonochemistry, and visible light/sunlight redox reactions.^26^ In the search for reactions that are speedy, efficient and take place under mild conditions, with high stereo- and enantio-specificity, much chemical ingenuity has been invested in catalyst design.^27^

### Benign by design

Beyond cleaner processes, sustainability requires paying much more attention to products that are ‘benign by design’ at all stages.^28^ An illustration can be seen in the case of ammonia, traditionally manufactured by the Haber–Bosch process which sources hydrogen by cracking CH_4_, as well as using energy to drive the reaction that is usually sourced from fossil fuels, together contributing massively to atmospheric release of CO_2_. While there are now major efforts^29^ to manufacture ‘green’ NH_3_, these need to be accompanied by great reduction in the excessive biogeochemical flow of nitrogen (Table 1). Other challenges for chemists include sustainable energy production from renewable, sustainable, non-polluting sources,^30^ including design of compounds that can mediate the transformation of solar energy to electricity;^31^ and capture of CO_2_ at the site of production^32^ as well as *via* atmospheric sequestration, combined with designing alternative fates for CO_2_,^33^ and developing sustainable alternatives to traditional concrete^34^ and reuse of waste concrete.^35^ Achieving goals for reductions in CO_2_ emissions requires analytical measurements and monitoring, integrated into comprehensive life cycle assessments, while design of alternative fates for CO_2_ must ensure that the carbon remains ‘locked up’ for long time periods and is not just briefly delayed on its journey back into the atmosphere.^36^ As noted by Artz *et al.*,^37^ this point requires a more nuanced approach to taking CO_2_ utilization as a measure for global CO_2_ mitigation, and the use of CO_2_ as feedstock for chemicals should be analysed as a route towards “de-fossilization”, rather than as a technology for CO_2_ mitigation.

### Circularity and the post-trash age

For chemists, imprints of the circular economy^38^ concept have been seen in ‘green chemistry’.^39^ With origins based in evolving industrial and academic movements, the term was defined and fleshed out as a set of 12 principles by Anastas and Warner.^40^ Green chemistry has had major impact, reorienting chemistry education, research and practice towards sustainability objectives.^39*a*,41^ Sustainable chemistry has many features in common with green chemistry, while emphasizing broader concerns with ecology, service and function and incorporating systems tools and transdisciplinary approaches.^42^ Similar principles are also seen in the more recent emergence of ‘circular’ chemistry^43^ which extended the 3Rs to 11Rs, transitioning to restorative and regenerative design principles for resource efficiency, utility and maintenance of high value when feasible.^44^ The concept of ‘one-world’ chemistry, which recognises the interconnectedness of the health of human beings, animals and the environment, adopts systems thinking and cross-disciplinary working to understand and navigate the interfaces between science, the environment and society and underscore chemistry's central position in providing the molecular basis of sustainability.^15*b*,45^ Embedded in all these concepts is the recognition that a new understanding is required of what has traditionally been termed ‘waste’ – the ‘unwanted’ materials that did not become incorporated into the intended product, as well as the product itself after its primary use. The World Bank estimated^46^ in 2018 that global generation of solid waste exceeded 2 billion tonnes per year and will rise by 70 per cent by 2050. The new understanding in the ‘post-trash’ age^47^ sees all the material outputs as resources for conservation and further reuse. This is especially critical when a process goes to scale and will determine whether it can deliver bulk and platform chemicals as well as large-volume speciality chemicals in a sustainable manner, with minimal environmental footprint and predictable re-uptake at the ‘end of life’ of the product. Achieving a post-trash society^47^ requires^48^ synergies at the interface of science, society and policy. The recent demonstration of computer-designed repurposing of waste to drugs^49^ signposts the directions of waste-to-valuable-resource reincarnation and circularity along which chemistry for sustainability should operate.

### Leveraging the digital transformation

Increasing use of the rapidly evolving digital tools and platforms, including big data handling, machine learning and artificial intelligence applications as well as robotics, is enabling chemists to make fundamental changes in their approach to sustainability challenges at all stages of the chemistry enterprise, including in Industry 4.0.^50^ These range from the scoping of the chemical space, which can rapidly identify large numbers of close analogues of any molecule to expand the chemical diversity of products under consideration,^51^ the prediction of chemical properties and design of optimum chemical pathways for production, including streamlining chemical explorations by application of machine learning to chemical reactivity,^52^ to the scoping of potentials for circularity options for recovery, reuse and recycling and the anticipation of likely environmental fates and ecological impacts of different pathways of sourcing, production, use and disposal. Across the spectrum, the chemist will be seeking to apply orientations that reduce the number of reaction steps, increase efficiency and recovery and minimise overall environmental impact, including reducing biogeochemical flows.

## Realigning chemistry and chemical industry for a sustainable world

The emergence of the ‘triple bottom line’ in business in the early 1990s added environmental and social concerns to the perennial focus on economics. While this extended the scope of business to include considerations of planet and people as well as profit, it is evident that a rebalancing among this trio of factors is now essential, with much greater priority being given to the planet component encompassing environmental protection and sustainability.^53^ The vital and urgent contributions needed from chemistry in response to the unfolding planetary boundaries crisis cannot simply happen within a ‘business as usual’ framework, and the present decade's worldwide political agreement on climate and sustainable development goals provides a narrow window of opportunity to avert catastrophic, long-term changes to planetary systems.^14*b*^ A dual-track response is required in which industry solutions that can be rapidly advanced and scaled up are given immediate support while, for the longer term, fundamental, systemic changes are progressively embedded to reorient the discipline as a leading sustainability science. These must reshape the way chemists approach all aspects of their work in education, research and industry.^15*b*,*c*^ It is essential to recognize that these three constituents have complementary roles, as demonstrated by the constructive ways that both academia and industry already serve as the sources and mediators of knowledge, processes and products and as contributors and observers in global policy processes within international bodies.

For industry, critical areas for attention will follow principles of decarbonising the economy and energy in general, including, for the chemical industry, decarbonising steps in manufacturing/synthesis, and cutting all process- and product-related environmental emissions.^54^ This entails paying attention to the sustainable sourcing of materials, green chemistry principles of manufacturing and in-built design to maximise material circularity. As an example, a German Chemical Industry Association study^55^ highlighted that achieving full greenhouse gas neutrality by 2050 will require heavy R&D investment in new process technologies for basic chemicals, and improved mechanical and chemical recycling processes, backed by a suitable policy framework and regulations. Replacement of fossil fuels as energy sources will necessitate developing processes to use electrons directly or *via* carriers such as hydrogen.

The science–society–policy interface is of critical importance in ensuring the real-world practicality of expectations for industry in relation to sustainability goals. In recent decades, there has been an increasing tendency for manufacturing and R&D operations to move to countries where they can be conducted at lower costs, where they may be subject to lower standards of environmental regulations and less rigorous compliance oversight. Rebalancing among the trio of factors in the triple bottom line can only be viable economically if there is a level playing field of regulatory factors. This requires global agreements on international standards for good manufacturing practice and environmental impacts. Combined efforts from UNEP, the World Trade Organization and the United Nations Conference on Trade and Development will be needed, with concerted inputs from chemistry and chemical industry through professional bodies and industry organizations. As well as the COP and SDG processes, the Montreal Protocol, finalised as an international treaty in 1987 and the Political Declaration and Implementation Plan adopted at the 2002 World Summit on Sustainable Development provide examples where the combined forces of science, industry, social demand and political policy have been focused on reaching agreement to achieve major environmental goals.^2,56^

Here we present three examples that are representative of different kinds of problems, to explore the implications for the discipline and profession of chemistry.

### New platforms for organic chemicals

Since the 19^th^ century, chemists have become accustomed to regarding hydrocarbon-related compounds as the main source of starting materials for synthesis. This has strongly influenced all aspects of chemistry: industry has come to depend on petroleum and natural gas for its feedstocks, with simple olefins, aromatics and their derivatives being among the largest-volume products manufactured;^57^ researchers have demonstrated their skill and ingenuity by developing processes for cracking, metathesising and functionalising hydrocarbons; and chemistry curricula and textbooks have traditionally reflected a mindset in which syntheses generally follow pathways of increasing functionalisation/oxidation from hydrocarbon precursors.

Ending the use of fossil hydrocarbons as fuels will eliminate one of the main drivers of climate change. In the short term, this will extend the availability of hydrocarbon raw materials for synthesis. However, in the longer term, chemistry must switch to renewably-sourced feedstocks for the ever-expanding range and scale of organic compounds required. The biological products of metabolism are the most likely source – and these are, overwhelmingly, highly oxidised compounds such as cellulose and lignin that require depolymerisation to serve as versatile feedstocks,^58^ as well as a host of other bio-molecules incorporating important features such as chiral centres and multiple functionalities. Implications for industry include the need to source bio-organic materials, not only cheaply and efficiently but in ways that do not compete with other critical areas such as natural habitats, food production and clean water, do not liberate environmentally-damaging wastes during transformations into products or subsequent use and disposal, but do offer practical avenues for material recovery, reuse or recycling in cyclical systems. The long-standing concept of ‘chemurgy’ (use of agriculture as source of chemical feedstocks) must now be revisited and revived, modernising the approach and bringing it into alignment with current understanding of sustainability principles and practices.^59^ A challenge for chemistry researchers is to switch from seeking regio- and stereo-selective oxidative processes for hydrocarbon functionalization to devising site-specific reduction and substitution reactions of highly functionalised backbones found in many biomolecules, while utilizing green, sustainable and circular chemistry principles and information on environmental requirements and impacts to guide the choices of reagents, solvents and conditions. Future chemistry graduates will need to be equipped with the knowledge and skills to take forward these industry and research ambitions, implying not only revised chemistry curricula, but also that competence is developed in systems thinking and cross-disciplinary working.

### Sustainability of plastics

Due to their ease of production, versatility and low cost, plastics emerged during the 20^th^ century as among the most ubiquitous and widely used materials manufactured on the planet – but with the majority of all the plastic ever made ending up in landfill sites or scattered in the environment.^60^ Deep concern about the multiple environmental impacts of plastics^22^ has been reflected in the passage of a historic resolution in the UN Environment Assembly, to forge an international legally binding agreement by 2024 that addresses the full life-cycle of plastics, the design of reusable and recyclable products and materials, and the need for enhanced international collaboration to facilitate access to technology, capacity building and scientific and technical cooperation.^61^ The challenge, “not only multidisciplinary but also inter- and transdisciplinary research with integrated and multifaceted approaches are needed to produce novel eco-friendly materials with features similar to those of traditional plastics, as well as with acceptable economic and environmental impact”, is clearly framed in a way that speaks to chemists. They will need to tailor their responses in ways that take account of the multiplicity of plastics for diverse applications, involving a broad range of chemical structures and functional groups. Redesign of plastics will be necessary, to supply a wide and growing range of applications while inventing novel structures that meet the new, urgent requirements for sustainability across the entire spectrum, from sourcing of monomers, through synthesis and uses, to return and further use. For short-term approaches, research has opened promising avenues related to the sourcing of suitable monomers from microorganisms and plants, the production of biodegradable and recyclable polymers with useful properties and the development of methods for the recovery of monomers at the end of the useful life of the material. The challenge for chemistry is a multi-faceted one, as illustrated by the case of microplastics (particles < 5 mm in size). These are either used as small beads in many applications in cleaning and cosmetics, or result from physical degradation of macroplastic products. They have become ubiquitous in the environment, even in deep-ocean and polar regions (microplastics have now been observed^62^ in fresh snow in the Antarctic), and have been identified as a major threat to ecological niches such as coral as well as to overall environmental stability. Chemists need to take a comprehensive, Earth-system approach to the design, production and fate of plastics to eliminate this environmental hazard, including through attention to biodegradation,^63^ recapture and reuse/recycling of plastic materials in green chemistry and circular processes, including strategies and processes for depolymerization.^64^

### Element stewardship

Every element in the periodic table with a stable isotope (as well as some radioactive ones) finds commercial applications^65^ and the number and diversity of these uses is continuously mushrooming. Moreover, of more than 60 elements that can be detected in at least trace amounts in the human body, at least 28 are thought to be required for aspects of human metabolism and most of the elements are utilized in pharmaceuticals and other medical products.^66^ Situated at the confluence of these manufacturing and health interests, chemistry's capacities to analyse, synthesise and design materials, complemented by increasing understanding about the interactions between chemicals and the environment, now define an emerging, critical role for chemistry: to be a leader in the stewardship of the planet's element resources. EuChemS has made important contributions to this objective through its work to highlight element scarcity and to engage on this issue at the science–policy interface.^67^

For a number of elements, low abundance levels and/or very restricted distribution of favourable extraction sites, combined with high importance in large-scale uses, has led to a focus on their ‘strategic’, ‘critical’ or ‘endangered’ character.^68^ Among prominent examples, lithium is receiving increasing attention due to its importance in batteries, making it a critical element for the global energy transition to electric-powered vehicles. With pressure on supplies from the known main sources, which are located in only a few countries (especially Argentina, Australia, Bolivia, Chile, China, and USA) and with a low substitutability at present, heightened attention is being given to new sources (including seawater) and Li substitutes, for example, batteries based on other metals such as Al, Au, Fe, Mg, Na, Zn.^69^ There is an urgent need to solve the battery problem for storing energy sustainably as the world moves towards decarbonised energy supplies. This example shows how element stewardship has multi-sectoral ramifications, ranging from policies to ensure access, as a strategic national, regional or geopolitical issue, to science and technology challenges linked with the efficiencies of extraction, use, recovery and recycling of available supplies and with the continual need to identify practical alternative materials to build resilience^70^ to threatened supply shortages.

For other, more highly abundant materials, elements stewardship must especially focus on concerns about the overall biogeochemical flows of anthropogenic materials and ways to recycle materials or return them to the environment with the least possible harm. The implications of chemistry principles that have long been the bedrock of the discipline – including that matter cannot be created or destroyed, but only transformed; and that entropy favours the increasing dispersal of both matter and energy – need to be understood also at a planetary scale. The dilution and dispersal of elements at large scale is both a waste of finite resources and a threat to the environment that supports all life on the planet, yet the avoidance of this waste also consumes energy and resources.

## Messages for the chemistry community

There have been alarms in the past, alerting the world to the growing dangers of increasing resource utilization, matter transformation and lack of attention to waste in all its forms, but these rarely resulted in effective action. This would have required major disruptions to economies and uncomfortable changes to patterns of personal, social and political behaviour. However, the latest alarm call, signalled by transgression of planetary boundaries and evidence that we are already at the brink of catastrophic long-term changes to Earth systems, is louder and more urgent. That the transgression of planetary boundaries is, in many cases, intimately connected with biogeochemical flows, makes the call of particular relevance to the profession of chemistry, which needs to contribute to practical technical solutions as well as carrying and amplifying the message at the science/society/policy interface. Fortunately, chemistry now has a better understanding of sustainability and more tools than ever with which to help achieve sustainable solutions – but it will need to undergo deep-seated changes, as highlighted here, to make the needed contributions.

Chemistry is vitally important for understanding and managing Earth's material and biogeochemical flows. Both conceptual and strategic shifts are needed in the design principles and planning of chemical transformations. These shifts necessitate extending the sphere of chemistry's concern beyond the conversion of resource to target, by adding the requirement that all material outputs will be further utilised, either by conversion to a new serviceable resource or by return to the cradle. Exemplifying the principles of circularity, the shifts must aim to maintain the utilitarian value of chemical matter for as long as possible. Beyond the issue of conservation of useful material, the conceptual and strategic shifts must also provide the bridge between chemistry and the critical determinants of sustainability and stability of Earth systems encapsulated in the planetary boundaries framework.

Stewardship of the planet's chemical resources is fundamental to efforts to reduce and, in future, prevent the transgression of planetary boundaries that presently threatens the stability of Earth systems on which all societies depend. The implications for chemistry involve reorientation of all aspects of the discipline and profession. In education, this includes a redirection of content towards green and sustainable chemistry,^35,45*a*^ coupled with the development of knowledge and competencies related to systems thinking, convergence working and biogeochemistry principles. In research, it includes awareness of the planetary boundaries and planetary impacts of biogeochemical flows, as well as the honing of skills in cross-disciplinary working and systems-based approaches. In industry, it includes a commitment to incorporate comprehensively the tools available, such as life-cycle assessments, circular material flow and absolute environmental sustainability criteria, into the development of processes and products that are benign and circular by design and avoid transgression of planetary boundaries.

## Author contributions

Conceptualization, S. A. M., S. E. C., A. K., H. H. and G. M.; investigation, S. A. M., S. E. C., A. K., H. H. and G. M.; project administration, S. A. M.; writing – original draft, S. A. M.; writing – review & editing, S. A. M., S. E. C., A. K., H. H. and G. M.

## Conflicts of interest

There are no conflicts to declare.

## Supplementary Material
